# Deep Learning for Early Breast Cancer Detection on Contrast-Enhanced Breast MRI: A Multicenter Study

**DOI:** 10.3390/cancers18142229

**Published:** 2026-07-11

**Authors:** Na Young Jung, Jihe Lim, Hyug-Gi Kim, Sung Hun Kim, Bobae Choi, Bo La Yun, Ga Eun Park, Jang-Hoon Oh, Bo Kyoung Seo, Min Sun Bae

**Affiliations:** 1 Department of Radiology, Eunpyeong St. Mary’s Hospital, College of Medicine, The Catholic University of Korea, Seoul 03312, Republic of Korea; nyjung1230@gmail.com; 2 Department of Radiology, Hallym University Dongtan Sacred Heart Hospital, Hwaseong 18450, Republic of Korea; itscindy21@hotmail.com; 3 Department of Radiology, Kyung Hee University Hospital, Seoul 02447, Republic of Korea; khyukgi@gmail.com (H.-G.K.); roineri5@gmail.com (J.-H.O.); 4 Department of Radiology, Seoul St. Mary’s Hospital, College of Medicine, The Catholic University of Korea, Seoul 06591, Republic of Korea; rad-ksh@catholic.ac.kr (S.H.K.); hoonhoony@naver.com (G.E.P.); 5 Department of Radiology, Chungnam National University Hospital, Daejeon 35015, Republic of Korea; med20@hanmail.net; 6 Department of Radiology, Seoul National University Bundang Hospital, Seongnam 13620, Republic of Korea; yunbola@gmail.com; 7 Department of Radiology, Korea University Ansan Hospital, Ansan 15355, Republic of Korea; seoboky@korea.ac.kr; 8 Department of Radiology, Korea University College of Medicine, Seoul 02841, Republic of Korea

**Keywords:** breast, carcinoma, magnetic resonance imaging, deep learning, artificial intelligence

## Abstract

**Background/Objectives**: Accurate visual categorization of small enhancing lesions on contrast-enhanced breast MRI remains challenging. The purpose of this study was to develop a deep learning (DL) model for detecting small (≤2 cm) invasive breast cancers using multi-institutional breast MRI data. **Methods**: This retrospective study included a total of 1721 women (mean age: 54 years; range, 21–87 years) with T1-stage invasive breast cancer across five hospitals. All patients underwent dynamic contrast-enhanced breast MRI before surgery between 2010 and 2020. Enhancing breast masses on early postcontrast T1-weighted images (14,917 labeled cancer images and 1443 labeled benign images) were used as the ground truth. In this multicenter cancer-enriched cohort, a DL model was developed to distinguish cancerous lesions from noncancerous lesions. MR images were independently reviewed by three breast imaging radiologists to detect subcentimeter (≤1 cm) cancers, and the readers were informed that each examination contained a single cancer. The performance of the model was evaluated using the area under the precision–recall curve (AUPRC), sensitivity, precision, and F1 score. **Results**: The DL model achieved an AUPRC of 0.42 in detecting small (≤2 cm) invasive breast cancers presenting as enhancing masses on MRI (sensitivity, 83.2%; precision, 33.2%; F1 score, 0.47). For subcentimeter (≤1 cm) cancers, the detection performance of the model was lower (sensitivity, 75.3%; precision, 20.8%; F1 score, 0.33) than that of the radiologists (mean sensitivity, 89.5%; mean precision, 72.9%; mean F1 score, 0.80). When the radiologists used the DL model, their mean precision in detecting subcentimeter invasive breast cancers improved from 72.9% to 83.2%, with no significant change in sensitivity (89.5% vs. 86.8%, *p* > 0.05). **Conclusions**: The DL model demonstrated potential as an assistive tool by improving radiologists’ precision for detecting small invasive breast cancers on contrast-enhanced breast MRI in this enriched reader-study setting, although it did not significantly improve their sensitivity.

## Simple Summary

Few studies have specifically evaluated artificial intelligence (AI) for detecting small invasive breast cancers on contrast-enhanced breast MRI. In this study, we included 1721 patients with single-site invasive breast cancer who underwent preoperative breast MRI. Datasets from three hospitals were randomly split into training (*n* = 1300, 76%) and validation (*n* = 91, 5%) sets, and datasets from two additional hospitals were used as an independent test set (*n* = 330, 19%). In addition, a reader study was performed using subcentimeter (≤1 cm) invasive breast cancers in the test set (*n* = 76). For subcentimeter (≤ 1 cm) invasive breast cancers presenting as enhancing masses on MRI, the deep learning (DL)-based detection model showed lower performance than breast imaging radiologists. With DL model assistance, radiologists achieved higher mean precision in detecting subcentimeter breast cancers, with no significant change in sensitivity.

## 1. Introduction

Breast magnetic resonance imaging (MRI) is the most sensitive imaging modality for breast cancer detection and has superior sensitivity compared with mammography, digital breast tomosynthesis, and ultrasound [1,2,3,4]. Given that abbreviated MRI protocols reduce scan time, interpretation time, and cost, there is growing interest in expanding supplemental screening with breast MRI, particularly for women with dense breasts or an intermediate risk of breast cancer [3,5,6]. Most breast cancers detected on screening MRI are invasive, smaller than 1 cm, and node negative [7]. However, a major limitation of MRI screening is its high false-positive rate.

In high-risk women who undergo screening with MRI and mammography, invasive cancers are more likely to be detected on MRI, whereas mammography is more effective at detecting ductal carcinoma in situ [7]. Furthermore, subcentimeter (≤1 cm) invasive cancers identified on MRI often exhibit benign imaging features, necessitating follow-up MRI to assess lesion growth [8]. MRI may yield both false-negative and false-positive results when used in a screening setting. Deep learning (DL) techniques have the potential to improve the accuracy of breast MRI, particularly for cancer detection [9,10,11,12].

While artificial intelligence (AI) systems have been successfully implemented in mammography, AI applications in breast MRI have evolved more slowly because of the complexity of MRI examinations and the limited availability of large datasets [13,14,15]. DL models have been proposed for cancer detection, classification, segmentation, and triage using breast MRI [9,10,11,12,16,17,18]. A recent literature review of 18 studies reported that the area under the curve (AUC) values of DL models for breast cancer detection on MRI ranged from 0.5 to 1.0, with other performance metrics, such as accuracy, sensitivity, and specificity, varying widely. The sample sizes in these studies were generally small, highlighting the need for further development and validation of AI-based systems for breast MRI.

To our knowledge, few studies have specifically investigated the use of AI for detecting small invasive breast cancers on breast MRI. Therefore, the purpose of this study was to develop and evaluate a DL model for computer-aided localization of early-stage invasive breast cancers using multicenter breast MRI data and to compare its performance with that of breast imaging radiologists. Rather than simulating a population-based screening, this study was designed as a proof-of-concept investigation to establish the feasibility of AI-assisted lesion detection before evaluation in more clinically representative screening and diagnostic settings.

## 2. Materials and Methods

This retrospective multicenter study was reviewed and approved by the institutional review board of the coordinating institution, with a waiver of informed consent. All the participating institutions obtained approval from their local institutional review boards. Data were collected using a cloud-based platform to facilitate secure aggregation across multiple centers.

### 2.1. Study Sample

**Figure 1:**
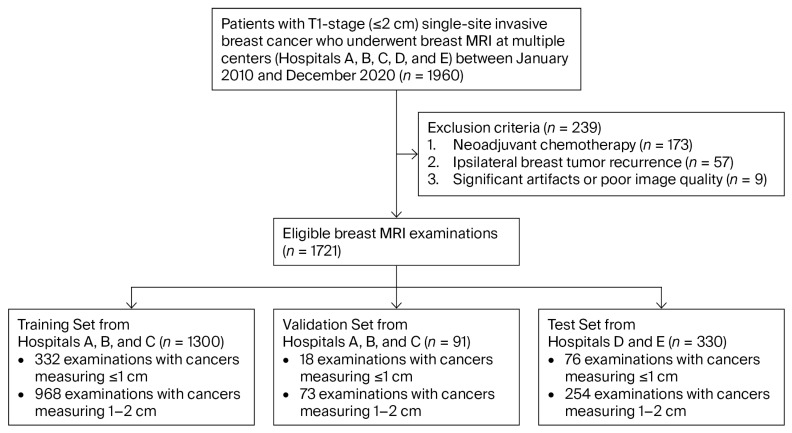
Flow diagram of patient eligibility and selection.

This study was conducted across five academic hospitals in South Korea. Radiology databases were queried to identify breast MRI examinations performed between January 2010 and December 2020 in patients with breast cancer who subsequently underwent surgery. The inclusion criteria were newly diagnosed invasive breast cancer presenting as an enhancing mass with or without associated non-mass enhancement (NME) on MRI and single-site invasive breast cancer 2 cm or less, as confirmed by surgical pathology. The exclusion criteria included the receipt of neoadjuvant chemotherapy, ipsilateral breast tumor recurrence, and significant artifacts or poor image quality, including post-biopsy changes (Figure 1).

### 2.2. Breast MRI Acquisition

MRI examinations were performed using 1.5- or 3-T systems with dedicated breast coils. The following systems were used: a 3-T Signa Architect (GE Healthcare) and a 1.5-T Signa HDxt (GE Healthcare) at Hospital A; a 3-T Verio (Siemens Healthcare) and a 3-T Ingenia (Philips Medical Systems) at Hospital B; a 3-T Ingenia Elition (Philips Medical Systems) and a 1.5-T Signa Excite (GE Healthcare) at Hospital C; a 3-T Achieva (Philips Medical Systems) at Hospital D; and a 3-T Skyra (Siemens Healthcare) at Hospital E. The MRI protocols included T2-weighted and dynamic contrast-enhanced T1-weighted sequences, comprising one precontrast and three or four postcontrast acquisitions. An intravenous gadolinium-based contrast agent (0.1 mmol per kilogram of body weight) was administered, immediately followed by a 20 mL saline flush. The detailed imaging protocols are provided in Supplementary Table S1.

### 2.3. Ground Truth Annotation

The datasets consisted of anonymized first postcontrast T1-weighted MR images from each institution. Ground-truth labeling was performed on these anonymized images using a MATLAB-based imaging tool (R2016b; MathWorks, Natick, MA, USA) for lesion-level annotation. Five fellowship-trained breast imaging radiologists (with 11 to 15 years of experience) and two radiology residents, under the guidance of a senior breast radiologist, drew rectangular bounding boxes around each enhancing mass and labeled them as malignant or benign according to the pathological results. The bounding boxes were drawn on a per-slice basis. The radiologists were assigned to label all cases from each institution, and they were all aware of the lesion pathology at the time of labeling. Among 1721 MRI examinations with a single malignant lesion, 178 (10.3%) also included concurrent benign lesions in either the ipsilateral or contralateral breast measuring ≤2 cm on MRI. Regarding benign lesions, the majority were pathologically confirmed, while the remaining lesions were considered benign based on imaging stability during more than 2 years of follow-up. The final annotated dataset consisted of 14,917 labeled malignant images and 1443 labeled benign images. Because this study included only patients with pathologically confirmed invasive breast cancer, normal (cancer-negative) MRI examinations were not included in the training, validation, or test cohorts.

### 2.4. Development of the DL Model

A cancer detection model was developed using the You Only Look Once (YOLO) architecture, initialized with pretrained SqueezeNet weights (Supplementary Figure S1). YOLO is a one-stage architecture that incorporates global contextual information, enabling rapid execution without compromising prediction accuracy in breast MRI [19,20]. The DL model was trained using the following hyperparameters: seven anchor boxes, the Adam optimizer, a mini-batch size of 128, an initial learning rate of 1 × 10^−3^, and an L2 regularization factor of 1 × 10^−4^. Training was conducted for up to 150 epochs. In this multicenter study, data aggregation revealed a significant class imbalance, with the cohort of verifiable benign lesions being substantially smaller than that of malignant lesions. Consequently, our training dataset primarily focused on pathologically confirmed malignant lesions.

Data augmentation techniques, including random rotations (90°, 180°, and 270°) and horizontal flipping, were employed during training to mitigate data scarcity and enhance model robustness. Image normalization was also performed as a preprocessing step. The seven anchor boxes were estimated by k-means clustering of the training-set bounding boxes using the intersection-over-union (IoU) metric as the distance measure the estimateAnchorBoxes function in MATLAB (R2019b; MathWorks, Natick, MA, USA). Seven anchor boxes were selected because the mean IoU reached a plateau, beyond which additional anchor boxes yielded only marginal improvement. The DL framework was implemented on Ubuntu 18.04 using MATLAB (R2019b; MathWorks, Natick, MA, USA), and all model training and inference were performed on an NVIDIA Quadro RTX 8000 graphics processing unit. During inference, candidate detections were filtered using a predefined confidence-score threshold and non-maximum suppression to retain the highest-confidence bounding box among overlapping detections. A retained detection that did not overlap a ground-truth bounding box with an IoU of at least 0.5 was considered a false-positive detection.

### 2.5. Reader Study

**Figure 2:**
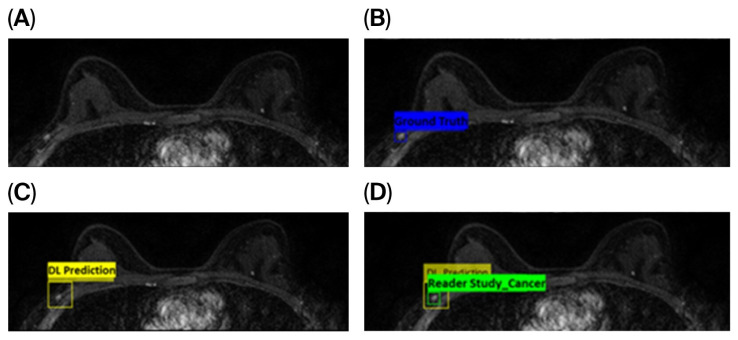
Reader study example of a screening-detected 0.6 cm invasive lobular carcinoma in a 47-year-old woman. (**A**) Early postcontrast T1-weighted MR image demonstrates an indistinct oval enhancing mass in the right upper outer breast. (**B**) The ground-truth annotation is shown as a blue bounding box. (**C**) The deep learning (DL) model localizes the lesion with a yellow bounding box. (**D**) In the reader study, all three radiologists identified the lesion as suspicious (green box). With DL assistance, two of the three radiologists assigned a higher Breast Imaging Reporting and Data System (BI-RADS) assessment category.

A retrospective reader study was conducted on subcentimeter (≤1 cm) breast cancers in the test set (*n* = 76). Three breast imaging radiologists (with 7–13 years of experience) who were not involved in the ground-truth labeling served as independent readers. A MATLAB-based imaging tool (R2016b; MathWorks, Natick, MA, USA) was used for lesion-level annotation during the reader study. The readers reviewed only the first postcontrast T1-weighted MR images, identified suspicious breast lesions by delineating regions of interest (ROIs) on each image slice, and assigned a probability of malignancy to each lesion based on the Breast Imaging Reporting and Data System (BI-RADS) [21]. The readers were aware that each MRI examination contained a single breast cancer but were blinded to its location. After a 4-week washout period, they repeated the image interpretation with AI assistance using the same MR images annotated by the DL model (Figure 2).

### 2.6. Statistical Analysis

The performance of the DL model for breast cancer detection was assessed using the area under the precision–recall curve (AUPRC), sensitivity, precision, F1 score, and the average number of false positives. Detection was performed on individual two-dimensional MR image slices; sensitivity, precision, and F1 score were calculated at the lesion-candidate (bounding-box) level, whereas the average number of false positives was reported per examination. Lesion localization performance was evaluated by comparing the predicted and ground-truth bounding boxes using an IoU threshold of 0.5. Performance metrics, including sensitivity and precision, are reported with 95% confidence intervals (CIs). Radiologist performance, with and without DL model assistance, was assessed using sensitivity, precision, F1 score, and the average number of false-positive detections. Pairwise comparisons of radiologist sensitivity with and without AI assistance were performed using McNemar’s exact test. All statistical analyses were performed using R software (version 4.0.1; R Foundation for Statistical Computing, Vienna, Austria), with statistical significance defined as *p* < 0.05.

## 3. Results

### 3.1. Study Population

A total of 1721 patients with single-site invasive breast cancer were included in the study. Datasets from three hospitals (A, B, and C) were randomly split into training (*n* = 1300, 76%) and validation (*n* = 91, 5%) sets, and datasets from two additional hospitals (D and E) were used as an independent test set (*n* = 330, 19%). Table 1 summarizes the patient and tumor characteristics for the training, validation, and test sets. The median ages of the patients in the training, validation, and test sets were 53 years (range, 21–87), 56 years (range, 31–80), and 53 years (range, 28–80), respectively. The training, validation, and test sets contained 332 (26%, 332 of 1300), 18 (20%, 18 of 91), and 76 (23%, 76 of 330) subcentimeter (≤1 cm) invasive breast cancers, respectively. The vast majority of tumors were invasive ductal carcinomas.

### 3.2. Performance of the DL Model

The DL model for breast cancer detection achieved an AUPRC of 0.42 on the test set. Table 2 summarizes the lesion-level performance. Detection performance was evaluated across three groups: subcentimeter (≤1 cm), 1–2 cm, and all cancers (≤2 cm). On the entire test set (*n* = 330), the DL model achieved a sensitivity of 83.2% (95% CI: 81.9, 84.4), a precision of 33.2% (95% CI: 32.9, 33.5), an F1 score of 0.47, and an average of 17.4 false positives. In the subcentimeter (≤1 cm) group, the sensitivity and precision were 75.3% (95% CI: 71.1, 79.3) and 20.8% (95% CI: 20.0, 21.7), respectively. Notably, the average number of false positives in this group was 3.9, which was lower than the number in the 1–2 cm group (13.4) or the overall cohort (17.4).

### 3.3. Radiologist Performance Using the DL Model

We evaluated the performance of radiologists in detecting subcentimeter (≤1 cm) breast cancers (*n* = 76) on MRI, both with and without DL model assistance (Table 3).

Overall, AI assistance slightly reduced the sensitivity (89.5% [95% CI: 84.7, 93.1] to 86.8% [95% CI: 81.8, 90.9]) but increased the precision (72.9% [95% CI: 72.0, 73.7] to 83.2% [95% CI: 82.5, 83.9]) compared with non-AI-assisted assessments; these differences were not statistically significant (*p* > 0.05). Two readers showed slight reductions in sensitivity with AI assistance (Radiologist 1: 88.2% [95% CI: 78.7, 94.4] to 81.6% [95% CI: 71.0, 89.5]; Radiologist 3: 92.1% [95% CI: 83.6, 97.0] to 90.8% [95% CI: 81.9, 96.2]), whereas Radiologist 2 showed no change in sensitivity (88.2% [95% CI, 78.7, 94.4]). All three readers demonstrated improved precision when using AI (Radiologist 1: 72.8% [95% CI: 71.2, 74.4] to 83.8% [95% CI: 82.3, 85.2]; Radiologist 2: 72.8% [95% CI, 71.2, 74.4] to 83.8% [95% CI: 82.6, 84.8]; Radiologist 3: 72.9% [95% CI: 71.6, 74.2] to 82.1% [95% CI: 81.1, 83.2]). The use of AI also led to a slight increase in the F1 score (0.80 vs. 0.85) and a reduction in the average number of false positives (0.33 vs. 0.18); however, these changes were not statistically significant (*p* > 0.05).

## 4. Discussion

Our study demonstrated that a DL-based MRI model could improve radiologists’ performance in detecting small invasive breast cancers. The DL model achieved an AUPRC of 0.42 (sensitivity, 83.2%; precision, 33.2%; F1 score, 0.47) for T1-stage breast cancers presenting as enhancing masses on MRI. For subcentimeter invasive cancers, the performance of the DL model was lower (sensitivity, 75.3%; precision, 20.8%; F1 score, 0.33) than that of the breast imaging radiologists (mean sensitivity, 89.5%; mean precision, 72.9%; mean F1 score, 0.80). When radiologists interpreted images with the assistance of the DL model, their mean precision improved from 72.9% to 83.2%, while their sensitivity remained similar (89.5% vs. 86.8%, *p* > 0.05).

MRI has the highest sensitivity for cancer detection among all breast imaging modalities; in fact, breast MRI can identify small tumors measuring less than 1 cm [7,8]. However, these small cancers may exhibit imaging features that overlap with those of benign lesions on dynamic contrast-enhanced MRI. Compared with larger cancers, subcentimeter invasive breast cancers more frequently demonstrate the benign-appearing characteristics of persistent enhancement and high T2 signal intensity [8]. Sung et al. compared the pathological features of breast cancers detected on screening breast MRI and those detected on screening mammography in high-risk women who underwent combined screening with those two modalities [7]. Their study reported that 82% of invasive cancers were diagnosed on breast MRI, whereas 65% of cancers detected on mammography were ductal carcinoma in situ. Previous studies have shown that the majority of breast cancers detected by MRI screening are invasive and smaller than 1 cm [2,3,4,7]. Our study highlights the potential of an AI-based MRI system to enhance radiologists’ performance in detecting subcentimeter (≤1 cm) breast cancers. AI-assisted MRI interpretation may facilitate accurate and efficient differentiation of small enhancing masses, potentially reducing false-positive results.

As the role of supplemental MRI expands in the screening setting, the use of abbreviated breast MRI is increasing because it maintains diagnostic accuracy while reducing imaging times compared with full-protocol breast MRI [5,22,23,24,25]. Because our DL model was developed using only the first postcontrast T1-weighted images, its design is compatible with the imaging input available in abbreviated breast MRI protocols. However, the present study was not designed to evaluate the performance of the model in an abbreviated MRI or screening setting. Moreover, our study population differed substantially from a true screening MRI population, as it consisted of a retrospective cancer-enriched cohort. Therefore, the present study should be regarded as a proof-of-concept investigation rather than a screening validation study.

The stand-alone performance of our model in the subcentimeter group was relatively low, likely because of the small sample size. The model performance slightly improved in the 1–2 cm group in terms of precision, sensitivity, and F1 score. The average number of false positives markedly decreased in the subcentimeter group compared with the 1–2 cm group (3.9 vs. 13.4). These findings align with those of prior studies showing that when enhancing breast masses exceed 1 cm in size, they more frequently exhibit malignant MRI features, such as irregular shape, non-circumscribed margins, and heterogeneous internal enhancement pattern [8,26,27]. Although detection performance in the subcentimeter group varied across readers and institutions, radiologists with AI assistance achieved better overall performance than either AI alone or radiologists alone in interpreting breast MRI. The mean precision and F1 score of the three radiologists tended to improve with AI assistance, and there was no significant decrease in sensitivity. The average number of false positives per examination also decreased with AI assistance (0.33 vs. 0.18). These findings suggest that a DL-based model for breast MRI may improve the performance of radiologists in detecting small breast cancers.

It is important to note that model performance varied across institutions. Several factors may have contributed to this variability, including differences in MRI acquisition protocols, scanner vendors, patient populations, and imaging characteristics. Furthermore, Hospital E contributed the fewest cases among the participating institutions, which may have resulted in less stable estimates of model performance. Nevertheless, in this multicenter study, the model was evaluated using an independent external test cohort collected from sites that were not included in model training, providing partial evidence of its generalizability across different institutions. Therefore, the findings of the present study should be interpreted with caution.

A major strength of this study is that the DL model was specifically developed to detect small invasive breast cancers on MRI, with the reader study focusing on subcentimeter lesions. The dataset was also larger than those of several previous studies, which included only a few hundred images [28,29,30,31,32]. To date, only one algorithm for lesion detection on breast MRI has been approved by the U.S. Food and Drug Administration [10]. Although the stand-alone detection performance of our model remains limited, particularly for subcentimeter cancers, the present study demonstrates that the feasibility of AI-assisted detection of small invasive breast cancers on MRI and provides a foundation for future model development. We anticipate that more advanced AI-based models for screening breast MRI will be developed and refined, ultimately improving diagnostic accuracy and clinical utility. In addition, AI-assisted breast MRI may have potential applications beyond screening and routine diagnosis, including the evaluation of patients presenting with carcinoma of unknown primary involving the breast [33].

Our study has several limitations. First, our training dataset primarily consisted of malignant lesions that met our inclusion criteria, whereas the number of concurrent benign lesions was substantially smaller. Introducing such a sparse benign class creates a long-tailed distribution, which is known to adversely affect the performance of convolutional neural networks [34,35]. Consequently, rather than risk model instability or overfitting to a limited and potentially non-representative set of benign lesions, we deliberately excluded benign labels during training and formulated the task as single-class detection of malignant lesions against background tissue. However, detecting malignant lesions against background tissue is fundamentally different from distinguishing malignant from benign enhancing findings. Because benign enhancing lesions were not included as a competing class during training, this design likely contributed to the relatively low precision and higher number of false positives per examination observed in our study. Second, the reader study evaluated lesion localization performance within a cancer-enriched dataset rather than clinical detection performance in a screening or routine diagnostic setting. This design may have increased reader sensitivity and may not accurately reflect the impact of AI assistance during routine breast MRI interpretation. Third, we used only the first postcontrast T1-weighted sequence for DL model development, because it represents the most essential component of dynamic contrast-enhanced breast MRI and is compatible with abbreviated breast MRI protocols. Incorporating additional MRI sequences, such as maximum intensity projection or T2-weighted images, may further improve model performance. Fourth, because no formal inter-radiologist agreement analysis or centralized calibration session was performed, annotation variability may have influenced lesion detection performance. However, all annotations were performed by experienced breast radiologists according to a standardized annotation protocol across the participating institutions. Finally, pure NME lesions were excluded, although masses with associated NME were included. The potential effects of background parenchymal enhancement were not specifically evaluated. In addition, the study did not include clinically representative screening examinations containing benign enhancing lesions or cancer-negative MRI examinations. Therefore, the generalizability of our findings to routine clinical practice remains uncertain, and prospective validation in clinically representative screening and diagnostic populations is warranted.

## 5. Conclusions

In conclusion, we developed a DL model for detecting early-stage invasive breast cancers on contrast-enhanced breast MRI. In this retrospective cancer-enriched cohort, the model demonstrated potential as an assistive tool by improving radiologists’ precision, although it did not significantly improve their sensitivity. The current model should be considered an investigational assistive tool rather than a system ready for routine clinical use. Further prospective studies in clinically representative screening and diagnostic populations, including benign enhancing lesions, pure NME lesions, and cancer-negative MRI examinations, are warranted to determine the clinical utility of AI-assisted breast MRI interpretation.

## Figures and Tables

**Table 1:** Characteristics of the Study Population.

Characteristic	Training	Validation	Test
No. of patients	1300	91	330
Age (years)			
Mean	53	56	54
Median	53	56	53
Range	21–87	31–80	28–80
Histologic type			
Invasive ductal	1256 (97%)	89 (98%)	317 (96%)
Invasive lobular or mixed	44 (3%)	2 (2%)	13 (4%)
Tumor size			
≤1 cm	332 (26%)	18 (20%)	76 (23%)
1–2 cm	968 (74%)	73 (80%)	254 (77%)
Hospitals	A, B, C	A, B, C	D, E
Period	2010–2020	2010–2020	2010–2020

**Table 2:** Lesion-level Detection Performance on the Test Set.

	Sensitivity (%)	Precision (%)	F1 Score	FPavg *
Hospital D (*n* = 248)				
≤1 cm	72.2 (67.3, 76.7)	29.6 (28.3, 30.9)	0.42	2.5
1–2 cm	82.8 (81.2, 84.4)	46.5 (46.1, 47.0)	0.60	8.7
All	81.4 (79.8, 82.8)	43.5 (43.0, 43.9)	0.57	11.2
Hospital E (*n* = 82)				
≤1 cm	88.5 (80.0, 94.4)	10.4 (9.7, 11.2)	0.19	8.0
1–2 cm	89.0 (86.5, 91.2)	21.9 (21.4, 22.3)	0.35	27.7
All	88.9 (86.6, 91.1)	19.5 (19.2, 19.9)	0.32	35.7
Hospitals D & E (*n* = 330)				
≤1 cm	75.3 (71.1, 79.3)	20.8 (20.0, 21.7)	0.33	3.9
1–2 cm	84.3 (83.0, 85.6)	36.1 (35.7, 36.4)	0.51	13.4
All	83.2 (81.9, 84.4)	33.2 (32.9, 33.5)	0.47	17.3

Note—Data in parentheses are 95% confidence intervals. * FPavg = the average number of false positives.

**Table 3:** Performance of AI and Radiologists Without and With AI Assistance in Detecting Subcentimeter (≤1 cm) Breast Cancer.

Indicator	AI	R1		R2		R3		R1–3	
		Alone	With AI	Alone	With AI	Alone	With AI	Alone	With AI
Hospital D (*n* = 61)									
Sensitivity (%)	72.2 (67.3, 76.7)	91.8 (81.9, 97.3)	86.9 (75.8, 94.2)	91.8 (81.9, 97.3)	90.2 (79.8, 96.3)	93.4 (84.1, 98.2)	91.8 (81.9, 97.3)	92.4 (87.5, 95.8)	89.6 (84.3, 93.6)
Precision (%)	29.6 (28.3, 30.9)	75.7 (74.3, 77.0)	85.5 (84.2, 86.7)	75.7 (74.3, 77.0)	85.9 (84.9, 86.9)	77.0 (75.8, 78.2)	84.9 (83.9, 85.8)	76.1 (75.4, 76.9)	85.4 (84.8, 86.0)
F1 score	0.42	0.83	0.86	0.83	0.88	0.84	0.88	0.83	0.87
FPavg	10.23	0.30	0.15	0.30	0.15	0.28	0.16	0.29	0.15
Hospital E (*n* = 15)									
Sensitivity (%)	88.5 (79.9, 94.3)	73.3 (44.9, 92.2)	60.0 (32.3, 83.7)	73.3 (44.9, 92.2)	80.0 (51.9, 95.7)	86.7 (59.5, 98.3)	86.7 (59.5, 98.3)	77.8 (62.9, 88.8)	75.6 (60.5, 87.1)
Precision (%)	10.4 (9.7, 11,1)	61.1 (53.7, 68.1)	75.0 (66.5, 81.9)	61.1 (53.7, 68.1)	75.0 (70.0, 79.4)	59.1 (54.2, 63.8)	72.2 (68.1, 76.0)	60.4 (56.6, 64.0)	73.9 (70.6, 77.0)
F1 score	0.19	0.67	0.67	0.67	0.77	0.70	0.79	0.68	0.75
FPavg	44.27	0.47	0.20	0.47	0.27	0.60	0.33	0.51	0.27
Hospitals D & E (*n* = 76)									
Sensitivity (%)	75.3 (71.1, 79.3)	88.2 (78.7, 94.4)	81.6 (71.0, 89.5)	88.2 (78.7, 94.4)	88.2 (78.7, 94.4)	92.1 (83.6, 97.0)	90.8 (81.9, 96.2)	89.5 (84.7, 93.1)	86.8 (81.8, 90.9)
Precision (%)	20.8 (20.0, 21.7)	72.8 (71.2, 74.4)	83.8 (82.3, 85.2)	72.8 (71.2, 74.4)	83.8 (82.6, 84.8)	72.9 (71.6, 74.2)	82.1 (81.1, 83.2)	72.9 (72.0, 73.7)	83.2 (82.5, 83.9)
F1 score	0.33	0.80	0.83	0.80	0.86	0.81	0.86	0.80	0.85
FPavg	19.22	0.33	0.16	0.33	0.17	0.34	0.20	0.33	0.18

Note—Data in parentheses are 95% confidence intervals. R = Radiologist, FPavg = the average number of false positives.

## Data Availability

The data presented in this study are available from the corresponding author upon reasonable request.

## Supplementary Materials

The following supporting information can be downloaded at: https://www.mdpi.com/article/10.3390/cancers18142229/s1, Table S1: Summary of MRI protocols; Figure S1: Schematic representation of the deep learning model development process using breast MRI.

## Abbreviations

The following abbreviations are used in this manuscript:

AI	Artificial intelligence
AUC	Area under the curve
AUPRC	Area under the precision-recall curve
BI-RADS	Breast Imaging Reporting and Data System
DL	Deep learning
NME	Non-mass enhancement
ROI	Region of interest
