# Supplementary material for: Deep Learning for Early Breast Cancer Detection on Contrast-Enhanced Breast MRI: A Multicenter Study
Source: Cancers (Basel). 2026 Jul 11;18(14):2229. doi: 10.3390/cancers18142229 (PMC13406529; doi:10.3390/cancers18142229)
Supplement: Supplementary file 1 [file cancers-18-02229-s001.zip › cancers-4394180-supplementary.pdf]

Supplemental Materials

Table S1. Summary of MRI protocols.

	Hospital A	Hospital B	Hospital C	Hospital D	Hospital E
Scanner name(s)	Signa Architect,<br>Signa HDxt	Verio, Ingenia	Signa Excite,<br>Ingenia Elition	Achieva	Skyra
Field strength	3.0 T, 1.5 T	3.0 T	1.5 T, 3.0 T	3.0 T	3.0 T
Breast coil	Yes	Yes	Yes	Yes	Yes
**T2-weighted imaging**					
Field of view (mm)	320~340	300~320	350~360	320	340
Matrix	320 × 256<br>320 × 224	576 × 403 484 ×<br>300	320 × 192<br>392 × 358	456 × 295	448 × 314
Slice thickness (mm)	3~4	4	4	3	4
Slice gap (mm)	0.3~0.4	0	0~0.3	0	0.4
Repetition time (ms)	8800~9800	3910~4530	3011~3516	2500	4850
Echo time (ms)	85~90	80~93	67~70	70	95
Flip angle (°)	90~110	80~90	90~100	90	80
**Contrast-enhanced T1-weighted imaging**					
Field of view (mm)	340~360	300	350~360	340	360
Matrix	300 × 252<br>320 × 320	332 × 332	340 × 340<br>357 × 359	340 × 226	512 × 358
Slice thickness (mm)	1~1.6	1.2~2	1	2	1.5
Slice gap (mm)	0	0	0	0	0
Repetition time (ms)	4.3~5.3	4.0~4.4	5~6.2	5.4	4.63
Echo time (ms)	1.6~2.1	1.7~1.8	1.95~3	2.7	1.67
Flip angle (°)	10	10~12	10~12	12	10

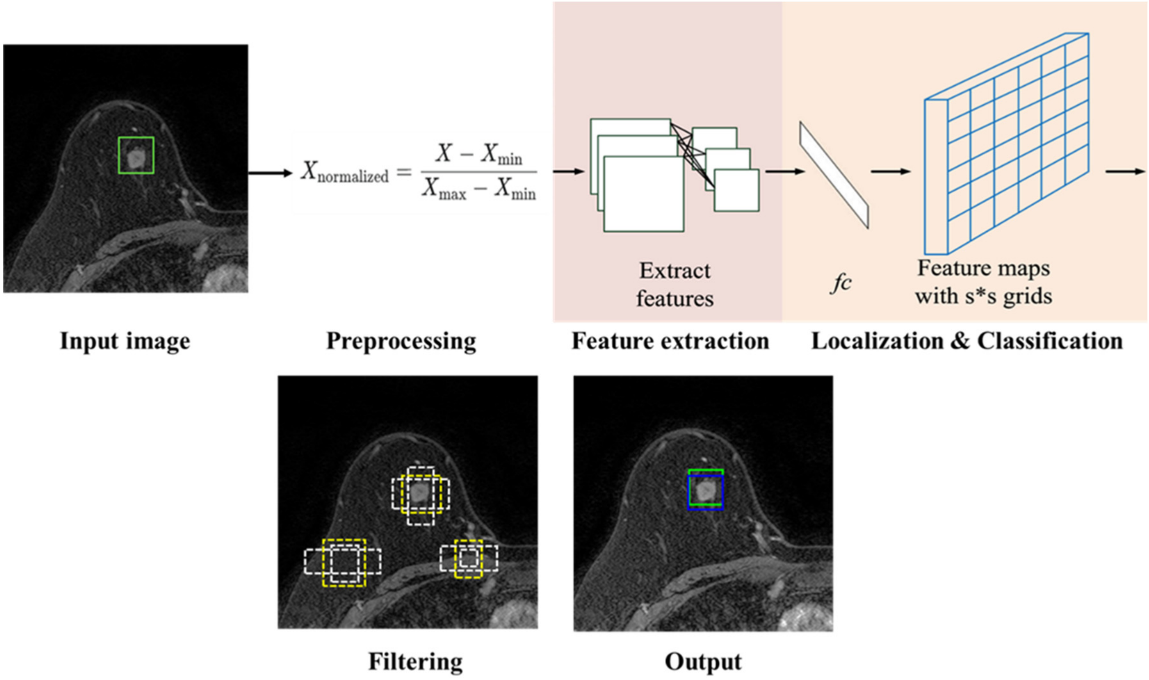

Figure S1. Schematic representation of the deep learning model development process using breast MRI.
